# Pathological mechanism and targeted drugs of ulcerative colitis: A review

**DOI:** 10.1097/MD.0000000000035020

**Published:** 2023-09-15

**Authors:** Meitong Guo, Xiaoyan Wang

**Affiliations:** a Changchun University of Chinese Medicine, Changchun City, China; b Jilin Academy of Chinese Medicine, Chaoyang District, China.

**Keywords:** pathogenesis, targeted drugs, UC

## Abstract

Ulcerative colitis (UC) is a chronic inflammatory disease of the colon with abdominal pain, diarrhea, and mucopurulent stools as the main symptoms. Its incidence is increasing worldwide, and traditional treatments have problems such as immunosuppression and metabolic disorders. In this article, the etiology and pathogenesis of ulcerative colitis are reviewed to clarify the targeted drugs of UC in the latest research. Our aim is to provide more ideas for the clinical treatment and new drug development of UC, mainly by analyzing and sorting out the relevant literature on PubMed, summarizing and finding that it is related to the main genetic, environmental, immune and other factors, and explaining its pathogenesis from the NF-κB pathway, PI3K/Akt signaling pathway, and JAK/STAT signaling pathway, and obtaining anti-TNF-α monoclonal antibodies, integrin antagonists, IL-12/IL-23 antagonists, novel UC-targeted drugs such as JAK inhibitors and SIP receptor agonists. We believe that rational selection of targeted drugs and formulation of the best dosing strategy under the comprehensive consideration of clinical evaluation is the best way to treat UC.

## 1. Introduction

Ulcerative colitis (UC) is a chronic idiopathic intestinal disease of unknown etiology that involves mainly the distal colon and rectum, often resulting in abdominal pain, diarrhea, and mucopurulent stools.^[1]^ UC is characterized by recurrent and remitting mucosal inflammation, and therefore, if not treated properly, leads to recurrent UC symptoms, ongoing intestinal damage, and increased risk of cancer.^[2]^ Nowadays, UC has become a global disease, and according to statistics, more than 1.5 and 2 million people in North America and Europe have suffered from UC in the past decade, and the incidence continues to increase, which means that the treatment of UC will pose a great challenge to health systems around the world.^[3,4]^ However, the etiology of UC is not fully understood and is now mostly thought to be related to various factors such as genetics,^[5]^ environment,^[6]^ infection,^[7]^ and immune regulation disorders,^[8]^ while damage to the intestinal mucosal barrier is central to the pathogenesis of UC. The intestinal mucosal barrier includes a mechanical barrier, an immune barrier, a biological barrier, and a chemical barrier, and the destabilization of any of the intestinal barrier functions will lead to the destruction of the intestinal mucosal tissue, which will cause inflammatory lesions.^[9,10]^

Current UC treatment still aims to induce and maintain remission of symptoms, reduce the risk of complications, and improve quality of life as the main goals.^[11]^ Individualized treatment plans are selected according to the severity of UC patients,^[12]^ and 3 major classes of drugs, namely aminosalicylic acid agents, glucocorticoids, and immunosuppressive agents, are mostly used clinically.^[2,6]^ Aminosalicylic acid agents can suppress the acute attack of UC and prolong the clinical remission period of UC patients, but in the subsequent treatment, aminosalicylic acid agents can cause gastrointestinal side effects such as loss of appetite, nausea, and vomiting, and have the possibility of autoimmune hemolysis, granulocytopenia, and nephrotoxicity.^[13]^ Glucocorticoids can regulate immune function and reduce inflammatory cell infiltration, thus suppressing the inflammatory response and inducing remission of UC disease, but glucocorticoids do not prevent the recurrence of UC and can lead to an early recurrence of UC when the dose is rapidly reduced, and serious adverse effects such as osteoporosis, muscle weakness, Cushing’s syndrome, growth inhibition, and peptic ulcers can occur with long-term use, so long-term use is not recommended.^[14–16]^ Immunosuppressants are mainly used in patients who are ineffective in aminosalicylate preparations or glucocorticoid therapy and toxic reactions to glucocorticoid therapy and long-term dependence on glucocorticoid use, but because immunosuppressants are cytotoxic, long-term use can cause serious adverse effects such as liver and kidney function impairment and bone marrow hematopoietic dysfunction, so they should also be selected with caution in clinical practice.^[17,18]^ With the application of monoclonal antibodies and recombinant proteins targeting cytokines and cytokines in the clinical treatment of UC, it marks a milestone in progress in the targeted treatment of UC. In this paper, we review the pathogenesis of UC and the research progress of UC-related targeted drugs.

## 2. Pathogenesis of UC

### 2.1. Genetic factors

UC patients have a certain genetic susceptibility, about 12% of UC patients have a family history,^[19]^ and there are racial differences, with the incidence rate of Caucasians being 3 times higher than that of blacks, and predominantly patients of Western and Northern European origin.^[20,21]^ UC can be induced by deletion, mutation, or overlap of genes on chromosomes.^[22]^ In addition, Luke Jostins^[23]^ analyzed all genome-wide association studies and immunoassay data from 75,000 Europeans and found that 163 loci were associated with confirmed inflammatory bowel disease (IBD), with 23 specific loci strongly associated with UC, but the study was only obtained in a cohort of European ancestry, which may be ethnically biased. Subsequently, a cross-descent association study of IBD was conducted with data from 86,640 Europeans in genome-wide association studies and immunoassay data and 9846 non-Europeans in immunoassay data, which identified 38 new risk loci, most of which were shared across ethnic groups and explained 8.2% of the risk of UC disease, and differences in allele frequencies (NOD2) or effect sizes (TNFSF15 and ATG16L1) or combinations of these factors (IL23R and IRGM) can lead to genetic heterogeneity in different populations.^[24,25]^ And by deeply resequencing 108 UC-associated candidate genes in the Korean population in search of novel variants, it was found that the genes BTNL2 and C5orf55 were associated with UC susceptibility or could be considered as susceptibility genes for the development of UC in Asian populations.^[26]^ Frauke Degenhardt^[27]^ identified through genetic association studies highly variable human Leukocyte antigen regions have been identified as important in UC susceptibility in different ethnic groups, while DRB1*01:03 is mainly found in Western European descendants and rare in non-Caucasians.

### 2.2. Environmental factors

Traditionally, UC is regarded as a Western disease, and the incidence of UC is concentrated in Western developed countries, but according to epidemiological studies, the prevalence of UC has increased nearly 20-fold in some Asian regions in the past 4 decades.^[28,29]^ This also suggests that environmental factors play a crucial role in the development of UC.^[30]^ The current study found that the environmental factors affecting the development of UC include: diet,^[31]^ smoking,^[32]^ and the use of antibiotics.^[33]^ With the improvement of people’s living conditions, the intake of high oil and fat, protein, and dairy products has increased, and the intake of dietary fiber has decreased,^[34]^ in which a large amount of intake of trans-unsaturated fat makes the risk of UC disease increased.^[35]^ Dietary fiber itself induces insulin resistance, regulates fatty acid imbalance caused by intestinal epithelial inflammation, alters intestinal permeability, promotes sulfate production, and alters the intestinal microbiota by promoting the growth environment of sulfate-reducing bacteria.^[36]^ Meanwhile, dietary fiber is metabolized into short-chain fatty acids by fermentation, inhibits the transcription of pro-inflammatory factors,^[37]^ and scavenges oxygen free radicals,^[38]^ so a high dietary fiber diet plays a preventive role in UC.

Smoking reduces the recurrence rate of UC by a mechanism related to the mediation of CO,^[39]^ and long-term exposure to CO can induce the production of heme oxidase-1, which represents an endogenous host defense mechanism with endogenous upregulation and effective cytoprotection against various pro-inflammatory stimuli and has powerful anti-inflammatory and antioxidant capacities.^[40,41]^ Studies have shown that the use of antibiotics in childhood can prevent the onset of UC,^[42]^ and patients with UC have immune dysfunction and have been attacked by bacteria, and the application of antibiotics has a certain therapeutic effect on UC-related intestinal infections,^[43]^ but the abuse of antibiotics can lead to changes in the intestinal microflora and cause immune system disorders to induce the onset of UC, so the rational use of antibiotics should be advocated in clinical practice.

### 2.3. Immune factors

The immune system plays an important role in the development of UC, and disruption of normal immune regulation in the gut or abnormal immune responses are important aspects of UC pathogenesis,^[44]^ which can be caused by pathological interactions between the microflora in the gut and mucosal immunity in genetically susceptible individuals.^[45]^ Neutrophils, macrophages, mast cells, T and B lymphocytes, and natural killer cells are involved in a continuous chronic immune process, and the loss of immune tolerance leads to inflammation due to the increased release of antibodies, cytokines, and pro-inflammatory mediators from these effector cells, which stimulate the proliferation of antigen-specific effectors, thereby triggering the adaptive immune system and leading to local and systemic inflammation.^[8]^ Th cells are also important effector cells in the intestinal immune response process, and in the normal state of the organism, Th1 and Th2 are in dynamic balance,^[46]^ while when external antigens invade the organism, Th1 cells are activated and Th2 cell function decreases accordingly, while Th2 cells expand in large numbers to stimulate increased secretion of cytokines and suppress Th1, which in turn suppresses the series of immune The Th2 cell function will be reduced, while the Th2 cell expansion will stimulate an increase in cytokine secretion and suppress Th1, which in turn will suppress a series of immune effects mediated by this cell.^[47]^ In patients with active UC, there is an imbalance between the constraints of Th1/Th2 cells in their colonic tissues, and inflammatory cells are heavily and continuously activated, aggregating and infiltrating the lining of the colon, where IL-1, IL-1 receptors, IL-6, IL-8, and TNF-γ are upregulated in the patients’ colonic tissues, with the most significant increase in TNF-γ and the IL-1/IL-1 receptor ratio.^[48]^ In contrast, for the exudative inflammatory response and epithelial ulcer-associated intestinal wall edema seen in UC patients, which is associated with an early cytokine IL-4 response, the IL-4 response decreases and is replaced by the Th2 cytokine IL-13 when the patient has a long duration of inflammation.^[49]^ It has also been suggested that innate lymphoid-like cells (ILCs), which are central to innate immunity, are at the surface of the intestinal mucosa, maintain intestinal homeostasis and play a role in fighting intestinal inflammation.^[50]^

### 2.4. Pathway mechanism

#### 2.4.1. NF-κB pathway.

NF-κB is an important intracellular nuclear transcription factor involved in the inflammatory response, immune response, regulation of apoptosis, and stress response of the body. In unstimulated cells, most NF-κB dimers are inactivated and retained in the cytoplasm by the binding of small inhibitory molecules from the family of NF-κB inhibitory proteins (inhibition of NF-κB).^[51]^ The NF-κB pathway is regulated by tumor necrosis factor (TNF)-α, interleukin (IL), etc. IKK (IKK) activation phosphorylates inhibition of NF-κB, degrades the proteasome, activates and releases NF-κB-related factor complexes into the nucleus, and activates the expression of target genes involved in cell proliferation and apoptosis, initiating downstream signaling pathways.^[52]^ It was found that the expression of intestinal mucosal inflammation was increased in both mouse models of ulcerative colitis and NF-κB P-p65 was abundant in colonic tissues, and the loss of regulatory factors on the NF-κB signaling pathway leads to pathological changes in the intestinal mucosa of UC, and the inflammation is further exacerbated by the stimulation of inflammatory factors that cause NF-κB to be activated, so the expression of NF-κB in colonic tissues also responds to the UC severity of the disease.^[53,54]^ HIF-1α, COX-2, IL-6, IL-1β, and TNF-α are influenced by NF-κB and induce and regulate the development of body immunity and inflammation through different pathways.^[55]^ HIF-1α can be produced in large amounts in hypoxic environments, regulates the neovascularization, and participates in several response processes such as inflammation,^[56]^ and UC The large number of immune cells aggregating at the site of intestinal inflammation creates a local hypoxic environment where HIF-1α is activated, which in turn promotes the synthesis of IL-10 and COX-2 and enhances the inflammatory response.^[57,58]^COX-2, the rate-limiting enzyme that catalyzes the synthesis of prostaglandins from arachidonic acid, is highly expressed in the UC mucosal epithelium and crypt, which can lead to an increase in prostaglandin E2,^[59]^ COX-2 expression is also regulated by the NF-κB pathway, and its expression is enhanced under conditions of tissue injury and inflammation, resulting in increased prostaglandins in UC patients, causing vasodilation, increased permeability, mucosal congestion and edema, and symptoms of abdominal pain and diarrhea.^[60]^ TNF-α, a proinflammatory factor mediating the pathogenesis of UC, is first seen in intestinal inflammation and promotes the C-reactive protein release, amplifying the inflammatory response, and can also affect vascular microcirculation by inducing apoptosis of intestinal epithelial cells, thereby inhibiting the repair of ulcerated surfaces in the intestine.^[61]^ IL-6 is at the center of the inflammatory factor burst along with TNF-α, and UC colonic tissue injury is accompanied by overexpression of these inflammatory factors, while IL-10 is an inflammatory suppressor with a significant inhibitory effect on inflammation in UC.^[62]^

#### 2.4.2. PI3K/Akt signaling pathway.

The PI3K/Akt signaling pathway is also involved in the regulation and release of inflammatory factors, and it can indirectly activate the transcription factor NF-κB through phosphorylated IKK, thus interconnecting with the NF-κB pathway to promote enhanced expression and secretion of inflammatory factors, leading to damage of the colonic mucosa.^[63]^ PI3K is a lipid second messenger of intracellular signaling,^[64]^ which consists of a catalytic structural domain P110 and a regulatory structural domain P85.^[65]^ Akt is a serine/threonine protein kinase encoded by the proto-oncogene c-akt and is a direct target protein of PI3K. PI3K can signal through tyrosine kinase-linked receptors or G protein-linked receptors,^[66]^ causing conformational changes in Akt, with simultaneous phosphorylation of the Ser473 site and Thr308 site, activating or inhibiting downstream signaling molecules, thereby regulating cell differentiation, proliferation, and apoptosis. TLR4 is a pattern recognition receptor with an important role in intestinal intrinsic immunity, recognizing lipopolysaccharide as a protein. TLR4 recognizes lipopolysaccharide and mediates transmembrane signaling,^[67]^ and is usually overexpressed in the colonic epithelium of UC patients. TLR4 induces intestinal inflammation mainly by recognizing pathogen surface-associated molecules to induce activation of downstream signaling pathways, which in turn activates the expression of inflammatory factors.^[68]^ TLR4 signaling activates the PI3K/Akt signaling pathway and causes downstream mTOR activation, and it was found^[69]^ that mTOR was aberrantly activated in colonic tissues of UC patients and DSS-induced UC mice, and mTOR inhibitors had good inhibitory effects on DSS-induced UC. PlGF (placental growth factor), as an angiogenic protein,^[70]^ is closely related to pathological angiogenesis and inflammatory responses in UC patients, and studies have confirmed that PlGF-induced angiogenic responses in UC patients are dependent on the migration and sprouting of HIMECs (human intestinal microvascular endothelial cells) in the PI3K/Akt signaling pathway,^[71]^ and that serum PlGF concentrations were significantly higher in UC patients compared to healthy controls,^[72,73]^ with increased vascularity occurring only in areas containing active inflammatory infiltrates and concomitant increased histopathology.^[74]^

In addition, patients with long-term chronic recurrent UC are at increased risk of carcinogenesis, and the PI3K/Akt signaling pathway is one of the pathways of inflammatory carcinogenesis and a key link in the development of UC to CRC (colorectal cancer).^[75]^ It has been suggested that IL23R variants in IBD may act as a protective variant or contribute to inflammation, and IL23R polymorphisms may also increase the risk of CRC.^[76]^ Razali et al^[77]^ detected 13 cytokine-induced somatic mutations in PI3K-related genes in long-standing UC, CAC, and CRC patients and found that most of these variants appeared in the IL23R, IL12Rß1, and IL12Rß2 genes, and the IL23R variant rs10889677, as a possible mutation,^[78]^ helps to gain insight into how the cytokine-induced PI3K pathway induces UC to develop into CRC, and it may be a breakthrough in the treatment of UC.

#### 2.4.3. JAK/STAT signaling pathway.

The JAK/STAT signaling pathway uses second messengers to transmit extracellular information to the nucleus, influencing target gene expression and cellular responses, coordinating intracellular signaling of more than 50 different ligands, and consisting of 3 parts: tyrosine-associated receptors, JAK/STAT, and the intracellular segment of tyrosine kinase-coupled tyrosine-associated receptors. It is believed that abnormal JAK/STAT signaling pathway can lead to abnormal t-cell differentiation as well as defective t-cell regulatory activity, which is an important mechanism in the pathogenesis of UC.^[79]^ JAK family (JAK1, JAK2, JAK3, TYK2) are non-covalently bound to cytokine receptors, while STATs, as substrates of JAK, can couple to tyrosine phosphorylation signaling pathway.^[80]^ Depending on the ligand and receptor, different combinations of JAK and STATs are highly specific activated to exert specific transcriptional regulation and mediate a variety of biological processes such as apoptosis, proliferation, differentiation, and migration of cells.^[81]^ The expression of cytokines (IL-6, IL-10, IFN-γ, IL-12, and IL-23) required for the maintenance of homeostasis in intestinal immune cells and stromal cells are mediated through the JAK/STAT signaling pathway,^[82]^ where IL-6 to gp130 receptors activate STAT3 through JAK1, JAK2, and TYK2,^[83]^ and IL-12 can stimulate JAK2 and TYK2 activity, leading mainly to STAT4 homodimer phosphorylation, and IL-23 activates mainly STAT3.^[84]^ Activation of STAT3 promotes pathogenic differentiation of Th17 cells and suppression of regulatory T cells, accelerating UC inflammation,^[85]^ and it was found that inhibition of STAT3 phosphorylation restores DSS-induced UC mice cellular homeostasis of Treg/Th17 in colonic tissue and alleviates the clinical symptoms of UC.^[86]^ Whereas JAK2 and TYK2-dependent STAT4 phosphorylation are only present in the transduction response of certain specific signals (e.g., IL-12R and IL-23R) in the gp130 receptor family,^[87]^ STAT4 phosphorylation is associated with Th1 activity and regulates IFN-γ expression, accelerating intestinal mucosal injury in UC, and studies have also confirmed that STAT4 levels in the cytoplasm of UC patients and increased levels of STAT4 phosphorylation in the nuclei of mucosal cells.^[88]^ And it was found that polymorphisms of single nucleotides containing JAK2, TYK2, STAT1, STAT3, and STAT4 genes increase the risk of developing UC.^[23]^

IL-27 and IL-35 are inhibitory cytokines that signal through JAK1 and JAK2 and play an important role in the immune regulation of UC,^[89]^ IL-27 induces the expression of IL-10 and also inhibits the expression of Th1, Th2, and Th17 cells,^[90]^ IL-35 downregulates the levels of TNF-α, IFN-γ pro-inflammatory cytokines and increase the response of Th1 and Th17 to exert anti-inflammatory effects to improve UC.^[91]^ Because the JAK/STAT pathway is such that JAKs can be linked to multiple STATs and STATs are the final effectors of signaling, studies explaining UC from a JAK family perspective are more limited. In previous studies, we can know that JAK1 and JAK3 are mainly expressed in T and B cells, JAK1-deficient mice die prenatally or perinatally,^[92]^ JAK2-deficient mice are mutationally lethal due to lack of erythropoiesis,^[93]^ and JAK3 knockout mice develop severe combined immunity that affects T and B cell development, resulting in intestinal epithelial cell differentiation defects and impaired intestinal barrier function, thereby increasing susceptibility to UC.^[94]^

## 3. UC molecular targeting drugs

With advances in drug development, the drug options for UC have become more diverse. The resulting targeted therapeutic agents include anti-TNF-α monoclonal antibodies, integrin antagonists, IL-12/IL-23 antagonists, JAK inhibitors, and SIP receptor agonists (Table 1). This article now introduces the mechanism of action of these drugs to bring thoughts to clinical UC drug selection.

**Table 1:** Targeted drugs for UC and characteristics of drug effects.

Type	Drugs	Characteristics of drug action
Anti-TNF-α monoclonal antibody	Infliximab (IFX)	Human-mouse chimeric IgG1 monoclonal antibody, targeting TNF-α, was administered intravenously.
	Adalimumab (ADA)	Fully human, recombinant IgG1 monoclonal antibody, targeting TNF-α, is better tolerated, especially in patients who have lost response to or are intolerant to IFX monoclonal antibody.
	Golimumab (GLM)	The fully humanized monoclonal antibody, targeting Tnf-α, has a much higher affinity for TNF than IFX and ADA. GLM is suitable for IFX - and ADA-refractory UC patients and can be injected subcutaneously.
Anti-integrin monoclonal antibodies	Vedolizumab	It specifically binds to α4β7 integrin and blocks the binding of α4β7 to MAdCAM-1.
	Etrolizumab	Bidirectional inhibition of α4β7 integrin and MAdCAM-1 and αEβ7 integrin and β7 subunit of E-cadherin.
	Natalizumab	Targeted blocking of the binding of α4β1 integrin to VCAM-1 and the binding of α4β7 integrin to MAdCAM-1 has the risk of PML.
IL-12/IL-23 antagonists	Ustekinumab	The fully human IgG1 monoclonal antibody targets the p40 subunit shared by both IL-12 and IL-23, thereby inhibiting IL-12 signaling and further activation of Th1 subsets of T cells, and blocking IL-23-mediated immune response and downstream activation of Th17 subsets of T cells.
	Risankizumab	Novel biological agents selectively target the p19 subunit of IL-23 and produce specific inhibition of IL-23 without interfering with the host immune response involved in IL-12. Phase II and III clinical trials are ongoing
	Brazikumab	
	Guselkumab	
JAK inhibitor	Tofacitinib	Inhibition of JAK1 and JAK3 reduces the activity of JAK signaling.
	Filgotinib	Inhibition of JAK1.
	Upadacitinib	There was a stronger inhibitory effect on JAK1.
SIP receptor agonists	Ozanimod	Selective binding of SIPR1 and SIPR5
	Etrasimod	Full agonist of S1PR1 and partial agonist of S1PR4 and S1PR5.

MAdCAM-1 = mucusmembrane address element cell adhesion molecule 1, PML = progressive multifocal leukoencephalopathy, TNF = tumor necrosis factor, UC = ulcerative colitis, VCAM-1 = vascular cell adhesion molecules.

### 3.1. Anti-TNF-α monoclonal antibody

TNF-α is produced by a subpopulation of immune cells in the gut of UC patients, associated with Th17 differentiation and involved in the regulation of innate and adaptive immunity. TNF-α mainly induces pro-inflammatory factors, and activates macrophages and T cells, causing epithelial cell damage and intestinal mucosal destruction.^[95]^ The anti-TNF-α monoclonal antibodies currently approved for UC treatment are mainly infliximab (IFX), adalimumab (ADA), and golimumab. Infliximab, as a TNF-α targeted drug, is a human-murine chimera IgG1 monoclonal antibody, which plays an important role in the treatment of UC, and the 2020 American Gastroenterology Association guidelines recommend it as a first-line drug to save acute severe UC.^[96]^ Animal experiments^[97]^have demonstrated that anti-TNF-α treatment improves UC activity, which may be related to the immune response triggered by IFX treatment and indirectly affects the intestinal flora. A prospective clinical study^[98,99]^showed that maintenance IFX treatment significantly reduced C-reactive protein and fecal calprotectin levels in UC patients and reduced the surgery rate from 27% to 11%.

ADA, a fully human, recombinant monoclonal IgG1 antibody injected subcutaneously, binds soluble and membrane-bound TNF-α, causing cell-mediated dependent cytotoxicity, repairing complement, and inducing T-cell apoptosis, thus acting as a therapeutic agent for UC.^[100]^ In a real-world study,^[101]^ adalimumab achieved clinical remission and mucosal healing in nearly one-third of patients with moderately to severely active UC within 52 weeks. And adalimumab was better tolerated, especially in patients who lost response or were intolerant to IFX monotherapy.^[102]^ The rate of clinical remission at week 8 was 16.5% in the ADA group and 9.3% in the placebo group (*P* < .05). The rate of mucosal healing at week 8 was 41.1% in the ADA group and 31.7% in the placebo group (*P* < .05).^[103]^

Godamumab (GDM), the third TNF-α drug approved for the treatment of UC, is synthesized from TNF-immunized transgenic mice. Studies have shown that GDM has a much higher affinity for TNF than IFX and ADA, and it is also superior to IFX and ADA in terms of conformational stability and inhibition of tumor necrosis factor-induced cytotoxicity.^[104]^ In a multicenter, randomized, double-blind, controlled study, the proportion of patients in clinical remission and mucosal healing at weeks 30 and 54 was higher in patients receiving 100 mg GDM (27.8% and 42.4%) than in those receiving placebo (15.6% and 26.6%; *P* = .004 and *P* = .002 respectively).^[105]^

### 3.2. Anti-integrin monoclonal antibodies

There are still patients who fail to achieve clinical improvement after treatment with TNF-α monoclonal antibodies or small molecule immunomodulators, which is related to patients’ insensitivity to the above drugs or drug off-target. UC patients have a large number of leukocyte aggregates in the intestinal tract after lesions occur, resulting in immune dysregulation in the intestine. Integrins are transmembrane receptors on the surface of leukocytes and consist of α and β subunits. α4β1 integrin mediates vascular cell adhesion molecules (VCAM-1) on gastrointestinal endothelial cells; Binding of α4β7 integrin to mucusmembrane address element cell adhesion molecule 1 (MAdCAM-1) of intestinal endothelial cells; αEβ7 integrin binds to E-calmodulin of mucosal epithelial cells,^[106]^ thus binding to memory T lymphocytes and blocking inflammation production, thus also being one of the ideal targets for UC therapy.^[107]^

Vedolizumab (VDZ) is a humanized IgG1 monoclonal antibody that selectively blocks the binding of α4β7 integrin to MAdCAM-1 of intestinal endothelial cells, reducing leukocyte adhesion to the intestinal epithelium and has high intestinal selectivity, which can better promote intestinal mucosal repair and thus effectively induce symptom relief in UC patients.^[108]^ In a 52-week-long clinical study of patients with active UC,^[109]^ 374 patients were given intravenous placebo at weeks 0 and 2, and 521 patients were treated with intravenous VDZ at weeks 0 and 2. During the DAI assessment at week 6, it was found that the response rate was 47.1% in the VDZ group and 25.5% in the placebo group (difference with adjustment for stratification factors, 21.7 percentage points; 95% confidence interval [CI], 11.6–31.7; *P* < .001). VDZ was significantly superior to placebo as an induction and maintenance treatment for UC.

Etrolizumab monoclonal antibody inhibits bidirectionally α4β7 integrin with MAdCAM-1 and αEβ7 integrin with the β7 subunit on epithelial calcium adhesion protein.^[110]^ In a phase III trial evaluating the efficacy and safety of etrolizumab in maintaining moderate to severely active UC remission, 31% of patients in the etrolizumab group achieved endoscopic remission at week 62 compared with 17% in the placebo group (*P* = .029 < 0.05) and was well tolerated in this population.^[111]^

In addition, Natalizumab, which targets the binding of α4β1 integrin to VCAM-1 and α4β7 integrin to MAdCAM-1, has a high clinical response rate, but the risk of progressive multifocal leukoencephalopathy,^[112]^ a serious and fatal adverse effect, has led to the gradual withdrawal of natalizumab from the market.

### 3.3. IL-12/IL-23 antagonists

IL-12 (composed of p40 and p35 subunits) and IL-23 (composed of p40 and p19 subunits) are pro-inflammatory factors produced by intestinal pathogens and are essential for the differentiation of CD4 naive cells.^[113]^ Ustekinumab (UST) is a fully human IgG-type monoclonal antibody that inhibits IL-12 signaling and further activation of the Th1 subpopulation of T cells by binding to the p40 subunit shared by IL-12 and IL-23, and, at the same time, blocks IL-23-mediated immune responses and activation of the Th17 subpopulation of downstream T cells. One study evaluated the efficacy of UST as induction and maintenance therapy in patients with moderate to severe UC,^[114]^ the remission rate was 38.4% when UST was used every 12 weeks and 43.8% when UST was used every 8 weeks. The effective rate was significantly higher than that of the placebo group (24.0%) (*P* = .002 and *P* < .001, respectively). In a real-world prospective study^[115]^ involving 95 UC patients, which was designed to evaluate the efficacy and safety of UST in the real world, 53% of UC patients responded at week 16.39% and 33% achieved remission at week 24 and week 52, respectively. In addition, the proportion of patients with elevated C-reactive protein and severe endoscopic activity in patients who achieved remission at week 16 was significantly lower than that in patients who did not achieve remission (52% vs 75%, *P* < .05, and 50% vs 74%, *P* < .05, respectively), suggesting that UST is effective in both short-and long-term treatment in real life.

Risankizumab, Brazikumab, and Guselkumab, as novel biologics, selectively target the p19 subunit of IL-23 and do not interfere with the host immune response in which IL-12 is involved, thus theoretically allowing for better efficacy. Phase II and Phase III clinical trials of these drugs are ongoing, and further studies are needed regarding their clinical efficacy, safety, advantages over other biologics, and optimal dosing strategies.^[116]^

### 3.4. JAK inhibitor

The JAK-STAT signaling pathway is involved in the regulation of innate and adaptive immunity and hematopoiesis, and it enables the production of cytokines by helper T cells and induces the onset of inflammatory responses in UC.^[117]^ Tofacitinib, a nonselective inhibitor of JAK, is the most widely used clinically as a non-immunogenic oral small molecule drug that mainly inhibits JAK1 and JAK3, and is now approved by the FDA and EMA for the treatment of moderate to severe UC.^[118]^ Animal and human organoid experiments^[119,120]^ found that Tofacitinib may exert clinical effects by reducing the activity of the JAK signaling pathway, disrupting the normalization of tight junction protein expression, correcting the function of PTPN2 in macrophages or intestinal epithelial cells, and restoring the integrity of the epithelial barrier. In the subsequent phase III trial to explore its efficacy, it was found that patients with moderate to severe UC who received Tofacitinib clinically had a remission rate of 16.6% at 8 weeks of induction therapy, which was significantly higher than that of 3.6% in the placebo group (*P* < .001). In the trial with 52-week maintenance therapy, 34.3% of the patients had remission at week 5, as compared with 11.1% of those in the placebo group (*P* < .001). A real-world study^[121]^ that included 260 UC patients found that 15.7% of UC patients experienced adverse reactions with Tofacitinib, with infection being the most common, 5.8% of the cohort reported serious adverse events, and 4.2% of the cohort required discontinuation of treatment. An analysis of clinical data from 1157 UC patients found^[122]^ that patients treated with Tofacitinib experienced reversible elevated lipid levels during dosing and that lipid levels were negatively correlated with high-sensitivity C-reactive protein levels, but cardiovascular adverse events occurred less frequently and were not related to the dose of Tofacitinib used.

Filgotinib, a second-generation selective JAK1 inhibitor, has been approved for UC treatment in Europe and Japan.^[123]^ In a randomized, double-blind, phase 2b/3 study,^[124]^ 26.1% of UC patients receiving 200mgFilgotinib achieved clinical remission at week 10, which was significantly higher than the remission rate of 15.3% in the placebo group (difference rate, 10.8%; 95% CI: 2.1–19.5, *P* = .0157). Regardless of experience with biologics, was effective in inducing and maintaining clinical remission, and Filgotinib was well tolerated, with a similar incidence of adverse events to the placebo group, with nasopharyngitis and headache being the most common.

Upadacitinib has a stronger inhibitory effect on JAK1. In a phase 2b trial,^[125]^ upadacitinib was used as induction therapy in patients with moderately to severely active UC, and at week 8, clinical responses were achieved in 5.19%, 6.7%, 5.15%, and 30.45% of patients receiving 8.5, 14, 3, and 13 mg, respectively. In contrast, patients who received a placebo did not have clinical remission (*P* = .052, *P* = .013, *P* = .011, and *P* = .002) and had the best efficacy at 45 mg of upadacitinib once daily. However, the incidence of herpes zoster was higher in patients using upadacitinib, and the study was not sufficient to assess the safety of upadacitinib due to the limitations of the study in terms of sample size, dose range, and duration of use. A subsequent multicenter, phase 3 study^[126]^ showed that the clinical remission rate at week 8 was 26% in UC patients receiving induction therapy with upadacitinib at a dose of 45 mg once daily, which was higher than the remission rate of 5% in the placebo group (*P* < .0001). The rate of clinical remission at week 52 was significantly higher with maintenance upadacitinib at a dose of 30 mg (52%) than with placebo (12%) (*P* < .0001), and induction and maintenance therapy was well tolerated, and no new safety risks were observed.

### 3.5. SIP receptor agonists

SIP is a membrane-derived lysophospholipid signaling molecule that specifically binds G protein-coupled receptors (SIPR1-SIPR5), and SIP receptor agonists target lymphocyte recirculation and block lymphocyte migration from lymphoid organs to the intestine along a chemotactic gradient, thereby effectively reducing intestinal inflammation,^[127]^ leading to the therapeutic drugs Ozanimod and Etrasimod. Ozanimod can optionally combine SIPR1 and SIPR5, and a phase 3, multicenter, randomized, double-blind controlled trial^[128]^ found that the clinical response rate of induction therapy at week 10 of Ozanimod (18.4%) was significantly higher than that of placebo group (6.0%) (*P* < .001), and the response rate of maintenance therapy at week 52 (37.0%) was significantly higher than that of the control group (18.5%) (*P* < .001). Endoscopic changes and mucosal healing were significantly improved, and the incidence of adverse effects was similar in the Ozanimod group and the placebo group, with elevated hepatic aminotransferases being the most common.

Etrasimod is a full agonist of S1PR1 and a partial agonist of S1PR4 and S1PR5, which controls the level of immune cells in the blood and reduces inflammation in the lining of the colon.^[129]^ In the phase 2 randomized trial of patients with moderately to severely active UC,^[130]^ 41.8% of UC patients in the Etrasimod2mg group had endoscopic improvement compared with 17.8% of those in the placebo group (*P* = .003). The primary and secondary clinical endpoints were met with Etrasimod 2 mg at week 12, with very few patients experiencing atrioventricular block. Further phase III trials of Etrasimod need to be conducted to clarify the safety and optimal treatment regimen of Etrasimod in UC.

## 4. Conclusion

The pathogenesis of UC is still unclear, and it is mainly related to genetic, environmental, immune, and related signaling pathway overexpression and activation, etc. Moreover, the pathogenesis of UC is not caused by a single factor, but by the interaction of multiple factors. Therefore, an in-depth investigation of the pathogenesis of UC and the analysis of the intrinsic connection of each factor can help to develop effective and safe new therapeutic drugs for UC. In the past decades, targeted drugs represented by TNF-α monoclonal antibodies have achieved certain therapeutic effects. With continuous research on the immune system, new therapeutic targets have been discovered, and drugs such as integrin antagonists, IL-12/IL-23 antagonists, and JAK inhibitors have shown better therapeutic effects. Novel therapeutic tools based on the pathogenesis of UC can be used as a breakthrough in the treatment of UC. Targeted therapies for UC have good clinical application prospects, but these drugs still have potential adverse effects, and real trials and long-term clinical data are needed to improve their safety and optimal dosing strategies in the treatment of UC.

## Author contributions

**Conceptualization:** Xiaoyan Wang.

**Data curation:** Meitong Guo.

**Writing – original draft:** Meitong Guo.

**Writing – review & editing:** Xiaoyan Wang.
